# Limnological data derived from high frequency monitoring buoys are asynchronous in a large lake

**DOI:** 10.1371/journal.pone.0314582

**Published:** 2025-03-06

**Authors:** Claire Stevens, Paul C. Frost, Nolan J. T. Pearce, James D. Kelley, Arthur Zastepa, Marguerite A. Xenopoulos

**Affiliations:** 1 Environmental and Life Sciences Graduate Program, Trent University, Peterborough, Ontario, Canada; 2 Department of Biology, Trent University, Peterborough, Ontario, Canada; 3 Environment and Climate Change Canada, Canada Centre for Inland Waters, Burlington, Ontario, Canada; INRA/Sorbonne University, FRANCE

## Abstract

Autonomous data collection is rapidly becoming an integral part of water quality monitoring, particularly for agencies looking to manage and protect aquatic ecosystems. While beneficial, it is unclear how the collection of these data can be applied in spatially complex large lakes (e.g., Laurentian Great Lakes) given the spatial heterogeneity of the ecosystem. To address this potential shortcoming in large lakes, we assessed the synchrony of sensor variables between 10 pairs of static buoys in the western basin of Lake Erie (western basin surface area =  3,282 km^2^). Within western Lake Erie, water temperature was highly synchronous whereas dissolved oxygen, turbidity, chlorophyll and phycocyanin were asynchronous. The extent of this asynchrony was higher with increasing spatial distance between buoys. We found that between pairs of static buoys, temperature, dissolved oxygen, and turbidity all experienced decreasing correlations with increasing distance. Our results show that if researchers intend to leverage these data to answer important questions and provide real-time applications related to environmental issues like harmful algal/cyanobacterial blooms, monitoring networks need to be designed carefully with spatial complexity in mind. While autonomous data collection has many benefits, the reliance on a single or limited network of anchored monitoring buoys in large lake ecosystems has a high probability of missing important spatial features of these systems.

## Introduction

Globally, freshwater ecosystems are experiencing degradation on an unprecedented scale due to pressures from multiple environmental stressors [1–3]. Given the widespread prevalence of environmental stressors, like nutrient pollution, invasive species, and global warming, there is a continuing need for robust monitoring efforts that allow researchers to disentangle the effects of these stressors and better predict future conditions. Ecological monitoring of lakes, which provide essential ecosystem services and support human life, is typically supported financially by governments, and decision makers must make strategic decisions on where to place these monetary resources to maximize the amount and usefulness of collected data.

Monitoring efforts (e.g., *in situ* water quality sampling) have traditionally had a prohibitive cost, in terms of funding, time, and labor, which are limited in their spatiotemporal coverage and resolution. However, advances in sensors, data storage, and real-time transmission have resulted in a rapid increase in the use of high-frequency monitoring (HFM) in limnology [4]. HFM networks provide a unique opportunity for real-time monitoring, and the information can be easily conveyed and interpreted by end users. While there are alternative methods like remote sensing and satellite imagery which, like HFM networks, offer large amounts of data, they typically require specialized skills that many end users do not have and can lack in temporal resolution. Furthermore, HFM networks can provide information which remote sensing cannot, like dissolved oxygen. Indeed, advances in computing power and sensor technology in recent decades led to improved capabilities to measure water quality parameters with greater frequency and at a lower cost than traditional sampling [5]. Most HFM sensors can collect data at a temporal resolution that was previously impossible, with sampling frequencies commonly ranging from < 1 to 15-minute intervals [4]. Even when considering the hidden costs of HFM networks, including the often-significant resources needed to clean and maintain sensors, this high temporal resolution captures previously unseen variability in the data, allowing for increased confidence in the measurements. In many cases, using autonomous sensors is also a safer and more pragmatic option than sampling *in-situ*, particularly during winter sampling, or when sampling in large lakes [6]. The convenience and capabilities of autonomous high-frequency measurements has led to HFM’s becoming an integral and common part of limnological research [4,7] and has pushed freshwater science and monitoring forward in the volume, velocity, variety, resolution, and relational nature of the collected data [4,8].

Despite providing data at unparalleled temporal resolutions, sensor-based monitoring requires substantial upfront investments into equipment and infrastructure, which results in a trade-off between the spatial and temporal resolution of the data collected. Indeed, limited spatial resolution sampling is a bias all too common in lake monitoring where lakes are typically studied in the vertical dimension (depth) and not horizontally across space [9]. However, lakes are spatially complex in three dimensions. Variable morphometry, bathymetry, and hydrology can result in spatial gradients in biological and chemical parameters within even relatively small lakes [10–12]. This spatial complexity is particularly evident in large lake ecosystems, where different regions of the same lake can vary significantly given strong currents and circulation patterns, the presence of multiple, large inflowing tributaries, and varying thermal dynamics. For example, the Laurentian Great Lakes experience spatial heterogeneity in fish [13,14], zooplankton [13,15,16], chlorophyll [17], nutrient chemistry [18,19], and physical conditions [20,21]. Thus, while HFM systems like anchored monitoring buoys (HFM sensors which are affixed to immobilized buoys), have numerous advantages, inferences made from sensor networks with limited spatial resolution could underestimate the complexity and variability of limnological variables within lake ecosystems.

Spatial synchrony is a phenomenon where temporal fluctuations in the variables of interest correlate between distinct spatial locations across large geographical distances [22,23]. Given this definition, an ecosystem with high synchrony among all monitoring locations would allow monitoring at a single location to fully capture the spatial complexity of the ecosystem. Conversely, low synchrony or asynchrony, when monitoring locations have highly dissimilar limnological conditions, would not allow for the ecosystem to be fully captured by low spatial resolution monitoring efforts. Spatial synchrony is related to the drivers of ecological phenomenon where more synchronous ecosystem variables tend to be driven by large scale external factors, and more asynchronous variables are driven by local scale factors [24,25]. For example, it is well understood that smaller scale internal biotic variables (e.g., phytoplankton) are often regulated by several abiotic factors, and that the complex interactions among these factors can prompt asynchrony across space and time [26–28]. Alternatively, some abiotic variables like temperature are often synchronous as they are regulated by large scale factors such as climatic forcing [24,26,29]. Many of the studies which propose this relationship between large scale, external processes causing synchrony are done among multiple small lakes, and not one large lake [25–27]. It is not well understood if this relationship will still be seen between different regions of one large lake; however, given the spatial heterogeneity and distinct regions observed within single large lake systems, a relationship between large scale external factors and synchrony may be found. This dichotomy between large- and small-scale processes adds a layer of complexity to the utility of monitoring sensor arrays, where different sensor network configurations are needed depending on the end goals of the monitoring efforts. Assessments of spatial synchrony, while ecologically informative, can also help to determine the level of spatial resolution needed to meet the objectives of monitoring programs. For example, by understanding the spatial synchrony of an ecosystem, redundancy between sampling locations can be mitigated and allow for a more efficient use of limited monitoring resources.

Here, we use a multi-institutional monitoring network in the western basin of Lake Erie to investigate the spatial synchrony of static buoy sensor data commonly used in limnology and management decision making. Lake Erie provides an ideal representative lake to assess the application of buoy monitoring networks, as recurrent nuisance and harmful cyanobacterial blooms within the western basin have prompted increased monitoring efforts with many different stakeholders (i.e., federal, state, provincial, and municipal governments, academic institutions, and water treatment plants) deploying monitoring buoys. In addition, Lake Erie is a large, complex lake ecosystem that is spatially heterogeneous both biologically and chemically. We hypothesize this physical and biochemical complexity will lead to asynchrony in biological and chemical parameters, including dissolved oxygen, chlorophyll, and turbidity. Whereas parameters driven by large scale, external processes, like water temperature will exhibit synchrony across the western basin.

## Methods

### Study area

**Fig 1:**
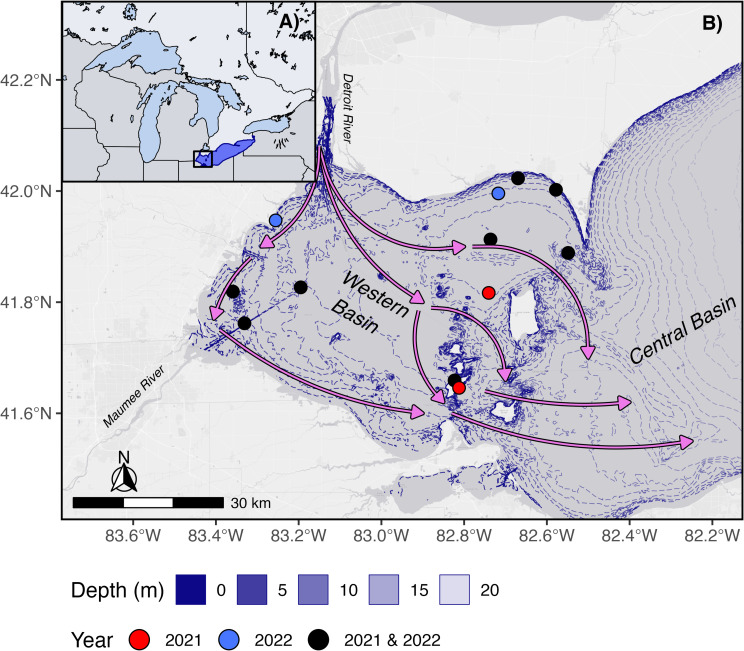
Map of the study area. (A) Lake Erie and the western basin in the Laurentian Great Lakes Region of North America, (B) deployed buoy locations in 2021 (red), 2022 (blue) and both years (black). Arrows represent typical prevailing circulation patterns in August in the western basin. Bathymetry data was acquired from NOAA [30].

Lake Erie (Fig 1) is the warmest, shallowest, and most biologically productive of the five Laurentian Great Lakes [31]. The western basin has an average depth of 7.4 meters, and the fetch of the entire lake is 388 kilometers. The western basin receives hydrochemical inputs from both the Maumee and Detroit Rivers. While most of the water volume is brought in by the Detroit River [32], the bulk of the nutrient load is derived from the Maumee River [33]. The circulation patterns are primarily driven by flow of the Detroit River but supplemented by wind forcing [32]. There can also be backflow from the central basin into the western basin, as well as anticyclonic gyres [34] creating unique complexities in circulation patterns. Additional biological complexity can be seen in the presence of invasive dreissenid mussels, *Cladophora* mats, and cyanobacterial harmful algal blooms (cHABs) [15,31].

Many anchored monitoring buoys have been deployed in Lake Erie, primarily with the intent of monitoring recurrent cHABs in the western basin. As Lake Erie is a source of drinking water for 11 million people [31], understanding the drivers and detecting the occurrence of these cHABs are top management priorities. Concerns surrounding cHABs has given rise to an increased monitoring effort in Lake Erie, with many different stakeholders deploying monitoring buoys.

### Data acquisition

Data for this study were acquired using the Seagull-GLOS (Great Lakes Observing System) platform and includes data from May-October in 2021 and 2022 collected via monitoring buoys operated by several different institutions (Table S1 in S1 File). The variables of interest for this study were all measured at the surface, and include temperature, dissolved oxygen, chlorophyll-a, phycocyanin, and turbidity. Additionally, Brunt-Väisälä frequency was calculated from surface and bottom water temperature measurements to represent vertical mixing using the ‘rLakeAnaylzer’ package in R [35]. Brunt-Väisälä values were only calculated from a subset of buoys that had temperature profile data available (n =  4; RAEON; Table S1 in S1: Table 1 in S1 File). To appropriately aggregate and standardize data across monitoring groups with various sensors deployed and given that only the overall trends (not the absolute value) of the data were of interest for this study, data from each buoy were separately z-score transformed prior to analysis, to standardize the relative scale across all buoys. Additionally, as different sensors collected data at varying temporal resolutions, all data were averaged on an hourly basis for consistency. Due to the large size of the dataset, interpolation for missing data was not done, and any missing observations were left blank.

### Synchrony analyses

Spatial synchrony in the time series of variables across monitoring buoys was assessed using three different methods: Pearson correlation, dynamic time warping, and Moran’s I.

#### Pearson correlation.

Using the R package ‘Ggally’ [36], a Pearson correlation coefficient was determined between the time series of all possible pairwise buoy combinations for each variable. The correlation coefficient was calculated for each hourly observation among all pairwise buoy combinations, then averaged to provide an overall measure of synchrony for each variable. Correlation coefficient was also calculated on a daily scale, to demonstrate temporal fluctuations in synchrony. Higher correlation coefficients indicate higher synchrony, and values closer to zero imply asynchrony. Additionally, the average correlation coefficient among all pairwise buoy combinations was calculated within daily time series to evaluate potential temporal trends in the degree of synchrony among monitoring locations for chlorophyll, phycocyanin, and dissolved oxygen. To address the role of circulation in synchrony, we paired wind speed data (aggregated using the ‘openmeteo’ R package [37]) with the daily Pearson correlation coefficient.

#### Dynamic time warping.

Dynamic Time Warping DTW is a bivariate algorithm which determines the optimal Euclidean distance between two time series [38]. DTW provides an advantage over using Pearson correlation alone, as DTW analysis is useful when comparing two time series of different lengths and can take into account consistently lagged but synchronous behavior. Despite this advantage, correlation analysis was still utilized, as it is used very commonly in synchrony analyses in ecology. Using the ‘dtw’ package in R, a normalized dynamic time warping value was calculated for all pairwise buoy combinations for each of the variables [39]. The normalized DTW distance is calculated by dividing the DTW distance by the length of the index and query time series. By using mean centered and scaled data, values are comparable among variables. A normalized distance of 0 indicates perfect synchrony (or no distance between the two time series), and increasing values imply decreasing synchrony. The DTW for all buoy combinations was averaged to provide an overall measure of synchrony for each variable. To visualize the relationship between the distance between buoys and synchrony, a linear regression was performed in the R package ‘ggplot2’ [40], with the distance as the predictor and either Pearson correlation coefficient or normalized DTW distance.

#### Moran’s Index.

Moran’s Index (MI) was used to check for spatial autocorrelation using the ‘ape’ package in R [41]. MI uses a weighted spatial matrix of coordinates to calculate spatial autocorrelation, where 0 implies no autocorrelation, and 1 and -1 indicate a perfectly dispersed or perfectly clustered dataset, respectively. The entire time series of each of the buoys was used as inputs for MI, yielding a single MI value for each variable.

In an analysis separate from the earlier mentioned regressions, non-parametric median-based linear regressions were used to model relationships between geographical distance (km) as a function of correlation for each variable, in the R package ‘mblm’ [42]. The models utilize Siegel’s repeated medians for estimating slope and significance is determined using non-parametric Wilcox Rank-Sum tests. This approach was chosen as statistical assumptions of a general linear model were not met for most variables; provided independence of observations, non-parametric models have no assumptions and median-based approaches are inherently robust to outliers [43]. The fitted models were then used to predict the geographical distance required between buoys to achieve a Pearson correlation coefficient of 0.5 (chosen because a coefficient of 0.5 is generally accepted as being a moderately strong correlation) for each variable in our study [44].

## Results

In 2021 and 2022, water temperature demonstrated the highest synchrony between pairs of buoys. Among all pairwise buoy combinations, Pearson correlation coefficients ranged from 0.89 to 0.99 in 2021 and 0.63 to 0.99 in 2022. This synchrony was also seen in temperature having the lowest average DTW of all variables in 2022, and the second lowest in 2021 (Table 1). Despite being spatially synchronous, temperature had a low Moran’s I, indicating no spatial autocorrelation. This, however, is an artifact of temperature being uniform throughout the basin. Brunt-Väisälä frequency was the next most synchronous variable. For Brunt-Väisälä frequency, r ranged from 0.28 to 0.85 in 2021, and 0.48 to 0.85 in 2022.

**Table 1:** Average Moran’s I (MI), Pearson correlation coefficient (r), and normalized dynamic time warping (DTW) distance for each high frequency monitoring (HFM) variable, averaged with all pairwise buoy combinations.

Year	Parameter	Temperature	Dissolved Oxygen	Turbidity	Chlorophyll	Phycocyanin	Brunt-Väisälä
2021	MI	-6.32×10^-5^	-3.66×10^-6^	-5.2×10^-6^	-2.62×10^-6^	-4.36×10^-4^	9.03×10^-7^
2021	r	0.96	0.39	0.18	0.18	0.10	0.45
2021	DTW	0.08	0.25	0.23	0.30	0.23	0.11
2022	MI	1.56×10^-3^^*^	2.72×10^-5^	-1.64×10^-4^	-4.96×10^-4^	-8.70×10^-4^^*^	-1.15×10^-2^^*^
2022	r	0.86	0.31	0.09	0.02	0.46	0.65
2022	DTW	0.09	0.23	0.15	0.19	0.19	0.12

* p <  0.05.

Dissolved oxygen, turbidity, chlorophyll, and phycocyanin were all asynchronous between pairs of buoys. Chlorophyll and phycocyanin displayed similar patterns. In 2021, chlorophyll had a DTW distance of 0.30, while phycocyanin had a DTW distance of 0.23. This asynchrony decreased in 2022, to DTW values of 0.19 for both chlorophyll and phycocyanin, respectively. Chlorophyll and phycocyanin also had the highest range of correlation coefficients of all parameters in 2021, with chlorophyll ranging from r =  -0.33 to r =  0.80, and phycocyanin ranging from r =  -0.62 to r =  0.76. This, however, was not true in 2022, where turbidity had the highest range of r =  -0.30 to r =  0.70. Turbidity was highly variable both spatially and temporally. The average r decreased from 0.18 to 0.09 from 2021 to 2022, and turbidity had a DTW distance range of 0.239 in 2021 and the second smallest range in 2022 (0.136). In 2021, chlorophyll had the highest DTW distance value of 0.30, whereas in 2022, dissolved oxygen had the highest DTW distance value (0.23). Excluding temperature and Brunt-Väisälä, phycocyanin and dissolved oxygen had the highest average correlation coefficients in 2021 and 2022 respectively (0.39 and 0.46). However, neither of these values were high enough to indicate strong synchrony. Dissolved oxygen, turbidity, chlorophyll, and phycocyanin all had MI values very close to zero, indicating no spatial autocorrelation.

### Temporal and spatial trends of synchrony

Pairwise correlation coefficients were significantly negatively correlated with distance between buoys for temperature (p_2022_ =  0.01, r_2022_ =  -0.39;), dissolved oxygen (p_2021_ =  0.004, r_2021_ =  -0.52; p_2022_ =  1.2×10^-4^, r_2022_ =  -0.59), turbidity (p_2022_ =  4.5×10^-6^, r_2022_ =  -0.62), and chlorophyll (p_2022_ =  1.4×10^-6^, r_2022_ =  -0.77. The correlation coefficient time series’ revealed that variables were generally more synchronous towards the beginning (May) and end (October) of the season. Turbidity, chlorophyll, and phycocyanin were typically the least synchronous during August and September. The pairwise normalized DTW values for temperature (p_2022_ =  2.08×10^-5^, r_2022_ =  0.58;), chlorophyll (p_2022_ =  3.52×10^-2^, r_2022_ =  0.39), and turbidity (p_2022_ =  3.95×10^-4^, r_2022_ =  0.51) were positively correlated with the distance between buoys in 2022, but not significantly correlated in 2021.

The linear model revealed that the average distance needed to achieve r =  0.5 for temperature was 108.10 km but varied between 2021 and 2022 (SD =  39.44). Dissolved oxygen required an average distance of 20.88 km (SD =  0.23) and turbidity required 24.34 km (SD =  16.73). The model was sigificant for chlorophyll in 2022 (p =  4.0 x 10^-6^) but not 2021 (p =  0.20). The average distance needed to achieve r =  0.5 for chlorophyll is 17.61 km, but this value varies between years (SD =  34.19). The model fit and significance was poor for phycocyanin for 2022 (p =  0.11), but less poor in 2021 (p =  0.05), where the predicted distance needed for r =  0.5 was 46.43 km. For both 2021 and 2022, the model fit was poor for Brunt-Väisälä (p_2021_ = 0.09, p_2022_ =  0.83).

## Discussion

High frequency measurements are an important and increasingly relied upon tool in limnological research [4]. Moored stations allow for a significant increase in the amount of data recorded [5]. However, sampling from a network static locations does not allow for robust spatial resolution. Indeed, we found asynchrony between pairs of static buoys in a multi-institutional network of 10 buoys across the western basin of Lake Erie - an area of 3,282 km^2^. Asynchrony was observed in the surface waters of the western basin of Lake Erie in dissolved oxygen, turbidity, chlorophyll, and phycocyanin, while surface temperature and Brunt-Väisälä frequency were synchronous. This asynchrony implies that while a spatially limited buoy network can derive useful information for the immediate area where they are deployed, these findings cannot capture the full complexity of a large lake ecosystem. Ideally, moorings that measure at depth in tandem with 3D hydrodynamic models and satellites would be needed to understand an ecosystem as large as the western basin of Lake Erie.

Consistent with synchrony literature, external climatic forcings such as air temperature and exposure to solar radiation, should be broadly consistent across all sensor locations, hence allowing for synchrony in water temperature readings between pairs of buoys. Our results suggest that water temperature from one sensor line would be relevant to most of the basin. While temperature loggers themselves are generally inexpensive, monitoring programs can benefit from this synchrony, as there is a significant amount of time and labor involved in sensor cleaning and maintenance. However, the overall goal of an HFM network should always be kept in mind, and if the goal was, for example, to specifically monitor fluctuations in temperature, we suggest multiple temperature sensors. Brunt-Väisälä frequency (a measure of stratification strength) is a function of water density and therefore linked to temperature, but was found to be less synchronous than surface water temperature. Additionally, the strength of the stratification in different regions of the basin is variable. For example, Brunt-Väisälä frequency at one buoy location was 6.96×10^-5^, while at the same time was 2.55×10^-3^ at a buoy 18.6 kilometers away. This variability in stratification strength is likely driven by the hydrological complexity [32,45] and the polymictic nature [46,47] of the western basin of Lake Erie.

Asynchrony in dissolved oxygen, turbidity, chlorophyll, and phycocyanin can be explained in part by the hydrodynamic patterns of Lake Erie. Lake Erie has complex circulation patterns (Fig 1B) and two major inflows (the Detroit and Maumee Rivers) which contribute to spatial heterogeneity [32,34,35]. Typically, from approximately May to September (the time of year when monitoring sensors were deployed), the strongest currents are in the northern side of the western basin [32]. The shallow (z =  7.4 m) western basin is polymictic, and is stratified roughly 60% of the time [46], but this stratification is weak. A windspeed of only 6 m s^-1^ is needed to vertically mix the western basin [47]. However, global warming has been shown to increase the strength and length of stratification [48], which may lead to future homogeneity in the western basin. The polymictic nature of the western basin could contribute to asynchrony, as stratification strength can vary across the basin, creating unique water masses. Conversely, frequently mixing could also drive synchrony (Figure S6 in S1 File), as mixing can more evenly distribute particulate matter throughout the basin. There are pathways in which hypoxic water can backflow from the central to the western basin and can contribute to the asynchrony of dissolved oxygen in the western basin [32,34,45]. There are also pathways which create water masses which flow opposite to the incoming Detroit River water, creating flows in opposing directions, likely contributing to asynchrony of dissolved oxygen [45]. It is likely that these backflows would also drive asynchrony in water temperature at depth, as the incoming hypoxic water masses would be expected to be colder than the relatively warmer waters of the western basin. These patterns of water movement indicate there are distinct water masses, each with varying amounts of chlorophyll, phycocyanin, and turbidity, circulating in the surface of the western basin. The movement of these water masses themselves are variable and, therefore, chlorophyll, phycocyanin, and turbidity between masses are variable as well. This variability may additionally explain why no spatial autocorrelation was detected despite being in a single lake. Additionally, the timing of the movement of the water masses likely contributes to the asynchrony detected between buoys.

**Fig 2:**
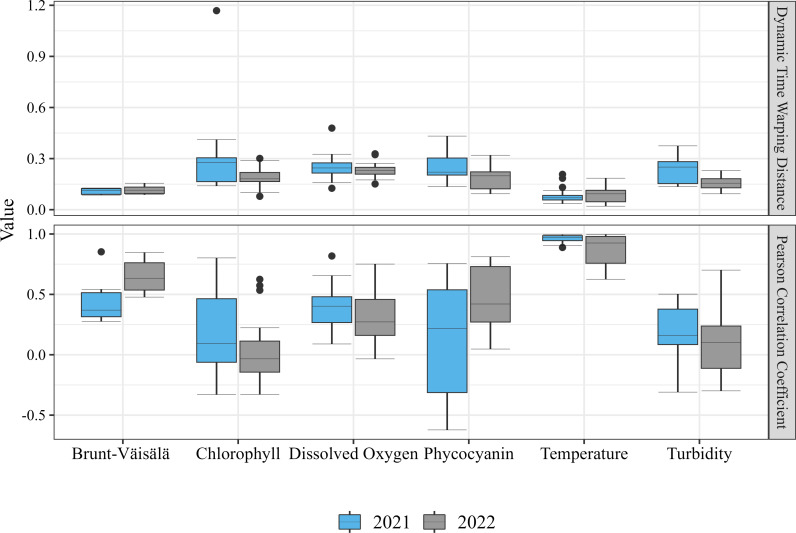
Boxplots created with all possible pairwise combinations of monitoring buoys in 2021 and 2022 for Pearson correlation coefficient and normalized DTW value.

**Fig 3:**
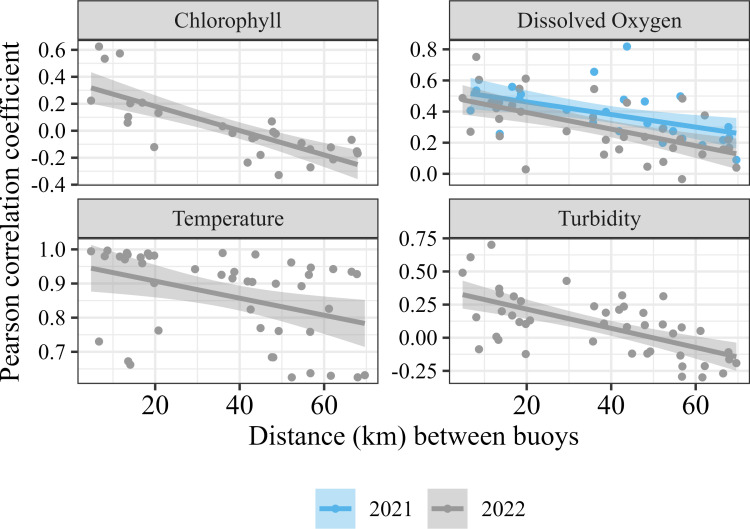
Linear regressions of the distance between buoys (km) and the Pearson correlation coefficient for each pairwise buoy combination for 2021 (blue) and 2022 (gray). Only significant (p <  0.05) trends are shown. Note that the scale of the y-axis differ between panels for clarity.

**Fig 4:**
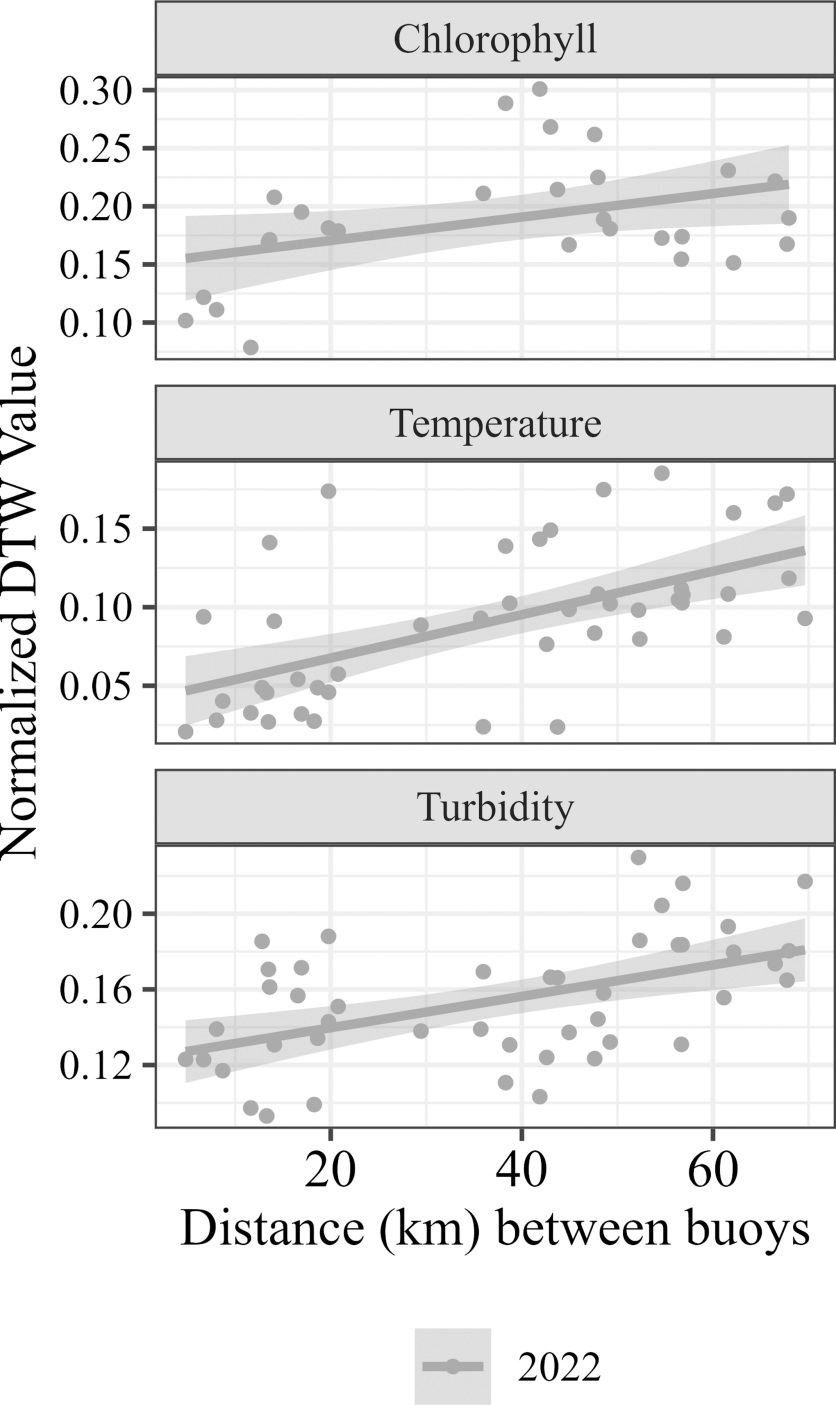
Linear regressions of the distance between buoys (km) and the normalized dynamic time warping value for each pairwise buoy combination for 2021 (blue) and 2022 (gray). Only significant (p <  0.05) trends are shown.

Asynchrony can also be explained by biological and chemical processes, like nutrient loading and patchy cyanobacterial/algal blooms. The western basin of Lake Erie is eutrophic, largely due to P-rich agricultural runoff [31,49,50]. This results in spatially large - yet possibly heterogenous patches of cHABs [49,51]. Blooms can be small enough that they encompass one sensor line, but not others [52–54], especially in more coastal nearshore areas. Thus, only one sensor line may capture the dissolved oxygen, chlorophyll, phycocyanin, and turbidity spikes from bloom growth, or oxygen depletion from bloom decomposition, while all other sensors are unaffected. The inputs of the Detroit and Maumee Rivers also contribute to internal drivers of heterogeneity, with each contributing unique nutrient loads, biotic communities, and water temperatures. This combination of biological and chemical factors as well as the hydrodynamics of the basin creates an ecosystem which is spatially asynchronous. Furthermore, the strength of the asynchrony detected varies temporally (Figs 2–4). For example, even though the overall trends are the same, the slopes of the lines in Figs 3 and 4 differ between 2021 and 2022, likely due to interannual variation in climate variables but also in biological and chemical processes. In Lake Erie, cHABs are typically most prevalent in late summer, which is also when turbidity, chlorophyll, and phycocyanin were all the least synchronous. This supports the idea that internal processes like cHABs can drive asynchrony in Lake Erie.

This asynchrony has both theoretical and practical applications for large lake ecosystems. The dynamics of the western basin of Lake Erie are complex, and asynchrony implies these dynamics are more driven by smaller scale, internal processes than larger scale, external processes [26,28]. Synchrony would indicate that the dynamics of the lake are primarily driven by external climatic conditions, in which the climate forces biological and chemical parameters to respond similarly. Conversely, asynchrony suggests there must be internal processes which act more strongly on biological and chemical parameters than external processes, causing spatial heterogeneity. These governing internal processes may include hypoxia leading to bioavailable P release from sediments [55,56], frequent and rapid stratification contributing to internal mixing [46], and the presence of invasive dreissenid mussels, *Cladophora* mats, and cHABs [31]. We acknowledge that this study only uses data collected at the surface, and in that regard it is difficult to investigate vertical processes; however, the western basin of Lake Erie is typically mixed [46], so it is possible that these vertical fluxes are captured by surface sensors. Practically, observed asynchrony in the western basin implies the current network of buoys does not capture the full complexity of the region. The full spatial complexity of the western basin of Lake Erie is likely being underestimated by buoys alone, and researchers should not rely solely upon anchored HFMs to draw conclusions about the lake.

### Implications of asynchrony for HFM networks

We found that the 10 buoys within the monitoring network did not capture the full spatial complexity of the western basin of Lake Erie, emphasizing that the locations in which monitoring resources are placed matters, even within a single lake, and that the placement of the resources depends largely upon the goals of the monitoring needs. For example, in the context of Lake Erie, the main intent of buoy placement in the western basin is to detect harmful cyanobacterial/algal blooms and safeguard drinking water from contamination. Thus, buoys are often placed in front of water intake valves. However, the findings of this study indicate that data from these buoys should not be used to draw conclusions about cHABs outside of the area which the buoys monitors. Additionally, these findings help demonstrate that an analysis of synchrony can aid decision-makers in determining where to optimally place monitoring buoys to best capture the full heterogeneity of the ecosystem. A synchrony analysis could be following a preliminary HFM buoy deployment to prevent placing monitoring buoys in redundant (synchronous) locations in following years and to optimize the information gained from a monitoring network.

### Potential for HFM deployment guidelines

We propose that the findings of Table 2 can be used as guideposts when deciding both where and how many HFM buoys to place in a large lake. We do however acknowledge that the findings of Table 2 do only represent one region of a single lake, and thus suggest that future synchrony analyses be done on other large lake ecosystems to build a comprehensive framework. Also, it is unreasonable to assume in the case of large lakes, one would have the resources to deploy enough buoys to capture the full complexity of the system. For example, to measure dissolved oxygen in the surface water of Lake Erie and fully account for spatial complexity, according to Table 2, our calculations indicate that at least 1,239 buoys are needed across Lake Erie. Since this is not feasible, the goals of the project should be considered to make strategic decisions on where buoys should be placed. If the goal of a project is to monitor temperature, fewer buoys would be needed to capture the spatial complexity of the ecosystem, when compared to something like chlorophyll or oxygen. It is also important to note that the distance we have calculated to find achieve synchrony is based on a goal correlation coefficient of 0.50. However, a different threshold could be chosen based on the desired goals on the monitoring array. For example, a desired threshold of r =  0.25 would imply increased distances needed between buoys, whereas a threshold of r =  0.75 implies decreased distances between buoys. This suggests a trade-off; one can invest less into buoys if you deploy fewer/farther apart, but there is a loss of spatial resolution. Additionally, our model has only been tested on one, very large lake, but we suggest that the direction of the relationship (i.e., increasing lake size meaning decreasing synchrony) is likely true for other types of lakes.

**Table 2:** Predicted distance (km) needed to achieve a correlation coefficient of at least r =  0.50 using the median-based linear regression models.

Year	Temperature	Dissolved Oxygen	Turbidity	Chlorophyll	Phycocyanin	Brunt-Väisälä
2021	135.40^*^	21.04^*^	36.17^*^	41.79	46.48	28.52
2022	80.21^*^	20.72^*^	12.05^*^	-6.57^*^	20.80	11.83

* p <  0.05.

Furthermore, most lakes are small, and have a surface area of 0.001 to 0.01 km^2^ [57], and our calculations assume that small lakes would only need one static buoy. For most parameters, multiple buoys would only be needed once a surface area of 10 km^2^ is exceeded (Table 2). Only 0.006% of the world’s lakes are 10 km^2^ or greater [57], although our calculations do not consider that smaller lakes are hydrodynamically less complex. In addition to lake size, the complexity of a lake should be considered when deciding how many buoys to deploy. While there is not currently a universal method for quantifying the complexity of a lake, we suggest here three main contributors to intra-lake complexity [11]. These considerations are 1) the thermal structure of a lake [21], 2) the presence of inflows [10], and 3) the presence of internal waves, turbulence, and intrusions [11].

In total, we propose three explanations for asynchrony between static buoys in large lakes. The first is that each static buoy is capturing distinct masses of water. The second is that there is a temporal lag between locations and water masses, and measuring variables at one point in time fails to account for the time it would take for a given water mass to travel from one static buoy to another. This would require greater in-depth knowledge of the complex circulation patterns and hydrodynamics of many large lakes which vary based on external drivers such as wind and heat. Lastly, the dynamics of internal versus external forcing cause differing responses between variables when measuring synchrony. Biological variables like chlorophyll are driven by both large-scale external and small-scale internal processes, which contribute to asynchrony in many systems not just large lakes. Whereas temperature is solely driven by large-scale external factors, leading to synchrony.

## Conclusions

More broadly, this study emphasizes that while anchored buoys are a useful tool, they lack in their ability to account for spatial heterogeneity and may underestimate the complexity of a system. Thus, monitoring efforts which utilize anchored monitoring arrays should be supplemented by other methods of data collection. Limnology has lagged behind oceanography, which has been aware of these potential shortcomings of static monitoring buoys and have noted the need for monitoring methods which can account for spatial variability [58]. These methods can include sampling *in-situ*, remote sensing, or using autonomous sampling techniques which have methods of propulsion (autonomous underwater vehicles, boat-mounted sensors, environmental sample processor, etc.). Additionally, these findings highlight the need to make strategic decisions about where anchored monitoring buoys are placed. There are always economic and logistical constraints regarding the number of buoys that can be deployed, so it is important to consider the spatial complexity of the study area, as well as the intended use of the monitoring array.

## Supporting information

S1 File **Table S1. Monitoring buoy information. Figure S1. Pearson Correlation matrices from May-October, 2021,** displaying temperature (A), dissolved oxygen (B), turbidity (C), chlorophyll (D), phycocyanin (E), and Brunt-Väisälä frequency (F). **Figure S2. Pearson Correlation matrices from May-October, 2022,** showing temperature (A), dissolved oxygen (B), turbidity (C), chlorophyll (D), phycocyanin (E), and Brunt-Väisälä frequency (F). **Figure S3. Matrices with normalized DTW distance from May-October, 2021**, showing temperature (A), dissolved oxygen (B), turbidity (C), chlorophyll (D), phycocyanin (E), and Brunt-Väisälä frequency (F). **Figure S4. Matrices with normalized DTW distance from May-October, 2022,** showing temperature (A), dissolved oxygen (B), turbidity (C), chlorophyll (D), phycocyanin (E), and Brunt-Väisälä frequency (F). **Figure S5. Daily correlation coefficient for limnological parameters in 2021 (blue) and 2022 (grey).** Correlations were calculated for each pairwise buoy combination then averaged to yield a final daily correlation value. **Figure S6. Linear regressions showing correlation as a function of wind speed in 2021 (blue) and 2022 (grey).**
(DOCX)

## References

[1] Albert JS, Destouni G, Duke-Sylvester SM, Magurran AE, Oberdorff T, Reis RE, et al. Scientists’ warning to humanity on the freshwater biodiversity crisis. Ambio. 2021;50(1):85–94. doi: 10.1007/s13280-020-01318-8 32040746 PMC7708569

[2] Birk S, Chapman D, Carvalho L, Spears BM, Andersen HE, Argillier C, et al. Impacts of multiple stressors on freshwater biota across spatial scales and ecosystems. Nat Ecol Evol. 2020;4(8):1060–8. doi: 10.1038/s41559-020-1216-4 32541802

[3] Jenny J-P, Francus P, Normandeau A, Lapointe F, Perga M-E, Ojala A, et al. Global spread of hypoxia in freshwater ecosystems during the last three centuries is caused by rising local human pressure. Glob Chang Biol. 2016;22(4):1481–9. doi: 10.1111/gcb.13193 26666217

[4] Meinson P, Idrizaj A, Nõges P, Nõges T, Laas A. Continuous and high-frequency measurements in limnology: history, applications, and future challenges. Vol. 24, Environmental Reviews. Canadian Science Publishing; 2015. p. 52–62.

[5] Hilbert M, López P. The world’s technological capacity to store, communicate, and compute information. Science. 2011;332(6025):60–5. doi: 10.1126/science.1200970 21310967

[6] Block BD, Denfeld BA, Stockwell JD, Flaim G, Grossart HF, Knoll LB, et al. The unique methodological challenges of winter limnology. . Vol. 17, Limnology and Oceanography: Methods. Wiley Blackwell; 2019. p. 42–57.

[7] Marcé R, George G, Buscarinu P, Deidda M, Dunalska J, de Eyto E, et al. Automatic high frequency monitoring for improved lake and reservoir management. Vol. 50, Environmental Science and Technology. American Chemical Society; 2016. p. 10780–94. 10.1021/acs.est.6b01604 27597444

[8] Kitchin R. Big data and human geography: Opportunities, challenges and risks. Dialogues Hum Geogr. 2013;3(3):262–7. doi: 10.1177/2043820613513388

[9] Crawford JT, Loken LC, Casson NJ, Smith C, Stone AG, Winslow LA. High-speed limnology: using advanced sensors to investigate spatial variability in biogeochemistry and hydrology. Environ Sci Technol. 2015;49(1):442–50. doi: 10.1021/es504773x 25406073

[10] Knauer K, Nepf HM, Hemond HF. The production of chemical heterogeneity in Upper Mystic Lake. Limnology & Oceanography. 2000;45(7):1647–54. doi: 10.4319/lo.2000.45.7.1647

[11] Kratz T, MacIntyre S, Webster K. Causes and consequences of spatial heterogeneity in lakes. In: Ecosystem function in heterogeneous landscape. Springer; 2006.p. 329–51.

[12] Rueda FJ, MacIntyre S. Flow paths and spatial heterogeneity of stream inflows in a small multibasin lake. Limnol Oceanogr. 2009;54(6):2041–57. doi: 10.4319/lo.2009.54.6.2041

[13] Harvey C, Kitchell J. A stable isotope evaluation of the structure and spatial heterogeneity of a Lake Superior food web. Canadian J Fisheries Aquatic Sci. 2000;57(1):1–10. doi: 10.1139/f00-123

[14] Scribner KT, Brenden TO, Elliott R, Donofrio M, Bott K, Kanefsy J, et al. Mixed stock analysis of genetic compositions of lake sturgeon (Acipenser fulvescens) mixtures in Lake Michigan: hierarchical spatial heterogeneity and evidence of improving recruitment in Wisconsin spawning populations. Can J Fish Aquat Sci. 2022;79(4):652–69. doi: 10.1139/cjfas-2021-0006

[15] Barbiero RP, Rockwell DC, Warren GJ, Tuchman ML. Changes in spring phytoplankton communities and nutrient dynamics in the eastern basin of Lake Erie since the invasion of Dreissena spp. Can J Fish Aquat Sci. 2006;63(7):1549–63. doi: 10.1139/f06-059

[16] Frost PC, Culver DA. Spatial and Temporal Variability of Phytoplankton and Zooplankton in Western Lake Erie. J Freshwater Ecol. 2001;16(3):435–43. doi: 10.1080/02705060.2001.9663833

[17] Stadig MH, Collingsworth PD, Lesht BM, Höök TO. Spatially heterogeneous trends in nearshore and offshore chlorophyll a concentrations in lakes Michigan and Huron (1998–2013). Freshwater Biol. 2019;65(3):366–78. doi: 10.1111/fwb.13430

[18] Neilson M, Stevens R. Spatial heterogeneity of nutrients and organic matter in Lake Ontario. Canadian J Fish Aquatic Sci. 1987;44:2192–203.

[19] Sterner R. The Laurentian Great Lakes: A biogeochemical test bed. Annual Review of Earth and Planetary Sciences. 2021;49(1):201–29. doi: 10.1146/annurev-earth-071420-051746

[20] Zhong Y, Notaro M, Vavrus SJ. Spatially variable warming of the Laurentian Great Lakes: an interaction of bathymetry and climate. Clim Dyn. 2018;52(9–10):5833–48. doi: 10.1007/s00382-018-4481-z

[21] Calamita E, Piccolroaz S, Majone B, Toffolon M. On the role of local depth and latitude on surface warming heterogeneity in the Laurentian Great Lakes. Inland Waters. 2021;11(2):208–22. doi: 10.1080/20442041.2021.1873698

[22] Buonaccorsi JP, Elkinton JS, Evans SR, Liebhold AM. Measuring and testing for spatial synchrony. Ecology. 2001;82(6):1668–79. doi: 10.1890/0012-9658(2001)082[1668:matfss]2.0.co;2

[23] Seybold EC, Fork ML, Braswell AE, Blaszczak JR, Fuller MR, Kaiser KE, et al. A Classification framework to assess ecological, biogeochemical, and hydrologic synchrony and asynchrony. Ecosystems. 2021;25:989–1005. doi: 10.1007/s10021-021-00700-1 36405421 PMC9671131

[24] Arnott SE, Keller B, Dillon PJ, Yan N, Paterson M, Findlay D. Using temporal coherence to determine the response to climate change in Boreal Shield lakes. Environ Monit Assess. 2003;88(1–3):365–88. doi: 10.1023/a:1025537628078 14570423

[25] Vogt RJ, Frost PC, Nienhuis S, Woolnough DA, Xenopoulos MA. The dual synchronizing influences of precipitation and land use on stream properties in a rapidly urbanizing watershed. Ecosphere. 2016 Sep;7(9).

[26] MAGNUSON JJ, BENSON BJ, KRATZ TK. Temporal coherence in the limnology of a suite of lakes in Wisconsin, U.S.A. Freshwater Biology. 1990;23(1):145–59. doi: 10.1111/j.1365-2427.1990.tb00259.x

[27] Rusak JA, Yan ND, Somers KM. Regional climatic drivers of synchronous zooplankton dynamics in north-temperate lakes. Can J Fish Aquat Sci. 2008;65(5):878–89. doi: 10.1139/f08-043

[28] Vogt RJ, Rusak JA, Patoine A, Leavitt PR. Differential effects of energy and mass influx on the landscape synchrony of lake ecosystems. Ecology. 2011;92(5):1104–14. doi: 10.1890/10-1846.1 21661571

[29] Benson BJ, Lenters JD, Magnuson JJ, Stubbs M, Kratz TK, Dillon PJ, et al. Regional coherence of climatic and lake thermal variables of four lake districts in the Upper Great Lakes Region of North America. Freshwater Biology. 2003;43(3):517–27.

[30] NOAA National Geophysical Data Center. Bathymetry of Lake Erie and Lake Saint Clair. NOAA National Centers for Environmental Information; 1999. doi: 10.7289/V5KS6PHK

[31] Watson SB, Miller C, Arhonditsis G, Boyer GL, Carmichael W, Charlton MN, et al. The re-eutrophication of Lake Erie: Harmful algal blooms and hypoxia. Harmful Algae. 2016;56:44–66. doi: 10.1016/j.hal.2016.04.010 28073496

[32] Beletsky D, Hawley N, Rao YR. Modeling summer circulation and thermal structure of Lake Erie. J Geophys Res Oceans. 2013;118(11):6238–52. doi: 10.1002/2013jc008854

[33] Stow CA, Cha Y, Johnson LT, Confesor R, Richards RP. Long-term and seasonal trend decomposition of Maumee River nutrient inputs to western Lake Erie. Environ Sci Technol. 2015;49(6):3392–400. doi: 10.1021/es5062648 25679045

[34] Beletsky D, Hawley N, Rao Y, Vanderploeg H, Beletsky R, Schwab D, et al. Summer thermal structure and anticyclonic circulation of Lake Erie. Geophysical Research Letters. 2012;39(6).

[35] Winslow M, Winslow L, Read J, Woolway R, Brentrup J, Leach T, et al. Package “rLakeAnalyzer” Title Lake Physics Tools [Internet]. 2022. Available from: https://github.com/GLEON/rLakeAnalyzer/issues

[36] Schloerke B, Cook D, Larmarange J, Briatte F, Marbach M, Thoen E, et al. GGally: Extension to “ggplot2”. 2021.

[37] Pisel T. openmeteo: Retrieve Weather Data from the Open-Meteo API. R package version 0.2.4. 2023. Available from: http://CRAN.R-project.org/package==openmeteo

[38] Bellman R, Kalaba R. On adaptive control processes. IRE Trans Automat Contr. 1959;4(2):1–9. doi: 10.1109/tac.1959.1104847

[39] Giorgino T. Computing and visualizing dynamic time warping alignments in R: The dtw package. Journal of Statistical Software. 2009.

[40] Wickham H. ggplot2: Elegant Graphics for Data Analysis. 2016.

[41] Paradis E, Schliep K. ape 5.0: an environment for modern phylogenetics and evolutionary analyses in R. Bioinformatics. 2019;35(3):526–8. doi: 10.1093/bioinformatics/bty633 30016406

[42] Komsta L. mlbm: Median-Based Linear Models. R package version 0.12.1. 2019.

[43] SIEGEL AF. Robust regression using repeated medians. Biometrika. 1982;69(1):242–4. doi: 10.1093/biomet/69.1.242

[44] Akoglu H. User’s guide to correlation coefficients. Turk J Emerg Med. 2018;18(3):91–3. doi: 10.1016/j.tjem.2018.08.001 30191186 PMC6107969

[45] Jabbari A, Ackerman JD, Boegman L, Zhao Y. Episodic hypoxia in the western basin of Lake Erie. Limnol Oceanography. 2019;64(5):2220–36. doi: 10.1002/lno.11180

[46] Loewen MR, Ackerman JD, Hamblin PF. Environmental implications of stratification and turbulent mixing in a shallow lake basin. Can J Fish Aquat Sci. 2007;64(1):43–57. doi: 10.1139/f06-165

[47] Boegman L, Loewen MR, Hamblin PF, Culver DA. Vertical mixing and weak stratification over zebra mussel colonies in western Lake Erie. Limnology & Oceanography. 2008;53(3):1093–110. doi: 10.4319/lo.2008.53.3.1093

[48] Kraemer BM, Anneville O, Chandra S, Dix M, Kuusisto E, Livingstone DM, et al. Morphometry and average temperature affect lake stratification responses to climate change. Geophysical Research Letters. 2015;42(12):4981–8. doi: 10.1002/2015gl064097

[49] Michalak AM, Anderson EJ, Beletsky D, Boland S, Bosch NS, Bridgeman TB, et al. Record-setting algal bloom in Lake Erie caused by agricultural and meteorological trends consistent with expected future conditions. Proc Natl Acad Sci U S A. 2013;110(16):6448–52. doi: 10.1073/pnas.1216006110 23576718 PMC3631662

[50] Scavia D, David Allan J, Arend KK, Bartell S, Beletsky D, Bosch NS, et al. Assessing and addressing the re-eutrophication of Lake Erie: Central basin hypoxia. Journal of Great Lakes Research. 2014;40(2):226–46. doi: 10.1016/j.jglr.2014.02.004

[51] Rowe M, Anderson E, Wynne T, Stumpf R, Fanslow D, Kijanka K, et al. Vertical distribution of buoyant Microcystis blooms in a Lagrangian particle tracking model for short-term forecasts in Lake Erie. Journal of Geophysical Research: Oceans. 2016;121(7):5296–314.

[52] Stumpf RP, Wynne TT, Baker DB, Fahnenstiel GL. Interannual variability of cyanobacterial blooms in Lake Erie. PLoS One. 2012;7(8):e42444. doi: 10.1371/journal.pone.0042444 22870327 PMC3409863

[53] Ho JC, Stumpf RP, Bridgeman TB, Michalak AM. Using Landsat to extend the historical record of lacustrine phytoplankton blooms: A Lake Erie case study. Remote Sensing of Environment. 2017;191:273–85. doi: 10.1016/j.rse.2016.12.013

[54] Manning NF, Wang Y-C, Long CM, Bertani I, Sayers MJ, Bosse KR, et al. Extending the forecast model: Predicting Western Lake Erie harmful algal blooms at multiple spatial scales. Journal of Great Lakes Research. 2019;45(3):587–95. doi: 10.1016/j.jglr.2019.03.004

[55] Nürnberg GK. Prediction of Phosphorus Release Rates from Total and Reductant-Soluble Phosphorus in Anoxic Lake Sediments. Can J Fish Aquat Sci. 1988;45(3):453–62. doi: 10.1139/f88-054

[56] Matisoff G, Kaltenberg EM, Steely RL, Hummel SK, Seo J, Gibbons KJ, et al. Internal loading of phosphorus in western Lake Erie. Journal of Great Lakes Research. 2016;42(4):775–88. doi: 10.1016/j.jglr.2016.04.004

[57] Downing JA, Prairie YT, Cole JJ, Duarte CM, Tranvik LJ, Striegl RG, et al. The global abundance and size distribution of lakes, ponds, and impoundments. Limnol Oceanogr. 2006;51(5):2388–97. doi: 10.4319/lo.2006.51.5.2388

[58] Lee C, Paluszkiewicz T, Rudnick D, Omand M, Todd R. Autonomous Instruments Significantly Expand Ocean Observing: An Introduction to the Special Issue on Autonomous and Lagrangian Platforms and Sensors (ALPS). Oceanography. 2017;30(2):15–7. doi: 10.5670/oceanog.2017.211

