# Supplementary material for: Limnological data derived from high frequency monitoring buoys are asynchronous in a large lake
Source: PLoS One. 2025 Mar 6;20(3):e0314582. doi: 10.1371/journal.pone.0314582 (PMC11884689; doi:10.1371/journal.pone.0314582)
Supplement: S1 File — Table S1. Monitoring buoy information. Figure S1. Pearson Correlation matrices from May-October, 2021, displaying temperature (A), dissolved oxygen (B), turbidity (C), chlorophyll (D), phycocyanin (E), and Brunt-Väisälä frequency (F). Figure S2. Pearson Correlation matrices from May-October, 2022, showing temperature (A), dissolved oxygen (B), turbidity (C), chlorophyll (D), phycocyanin (E), and Brunt-Väisälä frequency (F). Figure S3. Matrices with normalized DTW distance from May-October, 2021, showing temperature (A), dissolved oxygen (B), turbidity (C), chlorophyll (D), phycocyanin (E), and Brunt-Väisälä frequency (F). Figure S4. Matrices with normalized DTW distance from May-October, 2022, showing temperature (A), dissolved oxygen (B), turbidity (C), chlorophyll (D), phycocyanin (E), and Brunt-Väisälä frequency (F). Figure S5. Daily correlation coefficient for limnological parameters in 2021 (blue) and 2022 (grey). Correlations were calculated for each pairwise buoy combination then averaged to yield a final daily correlation value. Figure S6. Linear regressions showing correlation as a function of wind speed in 2021 (blue) and 2022 (grey). (DOCX) [file pone.0314582.s001.docx]

Limnological Data derived from High Frequency Monitoring Buoys are Asynchronous in a Large Lake

Claire. M. Stevens^1^, Paul. C Frost^2^, Nolan. J. T. Pearce^2^, James D. Kelley^1^, Arthur Zastepa^3^, Marguerite A. Xenopoulos^2^

^1^Environmental and Life Sciences Graduate Program, Trent University, Peterborough, Ontario, K9J 7B8, Canada

^2^Department of Biology, Trent University, Peterborough, Ontario, K9J 7B8, Canada

^3^Environment and Climate Change Canada, Canada Centre for Inland Waters, Burlington, Ontario, L7S 1A1, Canada

corresponding author: [clairestevens@trentu.ca](mailto:clairestevens@trentu.ca)

**Appendix S1**

**Table S1. Monitoring buoy information.**

| Buoy name | Buoy code | Operated by | GPS coordinate (Lat, Long) | Depth of water column | Distance to shore (km) | SensorDepth | Sensor brand | Frequency ofmeasurement | Temp. | Dissolved Oxygen | Chlorophyll | Phycocyanin | Turbidity | Years active |
| --- | --- | --- | --- | --- | --- | --- | --- | --- | --- | --- | --- | --- | --- | --- |
| Gibralter | GIB | Ohio State University | 41.65912, -82.8231 | 8.8 | 8.0 | Surface | YSI EXO2 | 15 min | K | % saturation | RFU | RFU | NTU | 2021, 2022 |
| GLERLWE2 | G2 | NOAA-GLERL | 41.762, -83.331 | 4.3 | 6.37 | Surface |  | 15 min | K | % saturation | RFU | RFU | NTU | 2021, 2022 |
| GLERLWE4 | G4 | NOAA-GLERL | 41.826667, -83.195 | 6.7 | 13.51 | Surface |  | 15-30min | K | % saturation | RFU | RFU | NTU | 2021, 2022 |
| GLERLWE8 | G8 | NOAA-GLERL | 41.819167, -83.3592 | 5.2 | 4.27 | Surface |  | 15 min | K | % saturation | RFU | RFU | NTU | 2021, 2022 |
| Monroe | MON | Wayne State University | 41.947181, -83.255645 | 2.7 |  | Surface |  | 10 min | K | - | RFU | RFU | NTU | 2022 |
| Put-in Bay | PUT | Village of Put-in Bay, Ohio | 41.3845, -82.4844 | 8.8 | 0.41 | Surface | YSI EXO3 | 20 min | K | - | RFU | RFU | NTU | 2021 |
| RAEON1 | R1 | University of Windsor (RAEON) | 41.8559670, -82.727200 | 10.1 | 17.2 | Surface, Bottom | Turner, Innovasea | 10 min | C | - | - | - | - | 2021 |
| RAEON2 | R2 | University of Windsor (RAEON) | 41.913017, -82.7367 | 10.4 | 11.16 | Surface, Bottom | Turner, Innovasea | 10 min | C | % saturation | - | - | NTU | 2021, 2022 |
| RAEON4 | R4 | University of Windsor (RAEON) | 41.8927, -82.5258 | 10.1 | 2.26 | Surface, Bottom | Turner, Innovasea | 10 min | C | % saturation | - | - | NTU | 2021, 2022 |
| RAEON5 | R5 | University of Windsor (RAEON) | 42.002583, -82.5786 | 6.7 | 0.89 | Surface, Bottom | Turner, Innovasea | 10 min | C | % saturation | ug/L | - | NTU | 2021, 2022 |
| RAEON6 | R6 | University of Windsor (RAEON) | 42.0222, -82.6697 | 6.7 | 1.58 | Surface, Bottom | Turner, Innovasea | 10 min | C | % saturation | ug/L | - | NTU | 2021, 2022 |
| RAEON7 | R7 | University of Windsor (RAEON) | 41.99605, -82.7179 | 8.8 | 3.25 | Surface, Bottom | Innovasea aquaMeasure | 10 min | C | % saturation | ug/L | ug/L | NTU | 2022 |


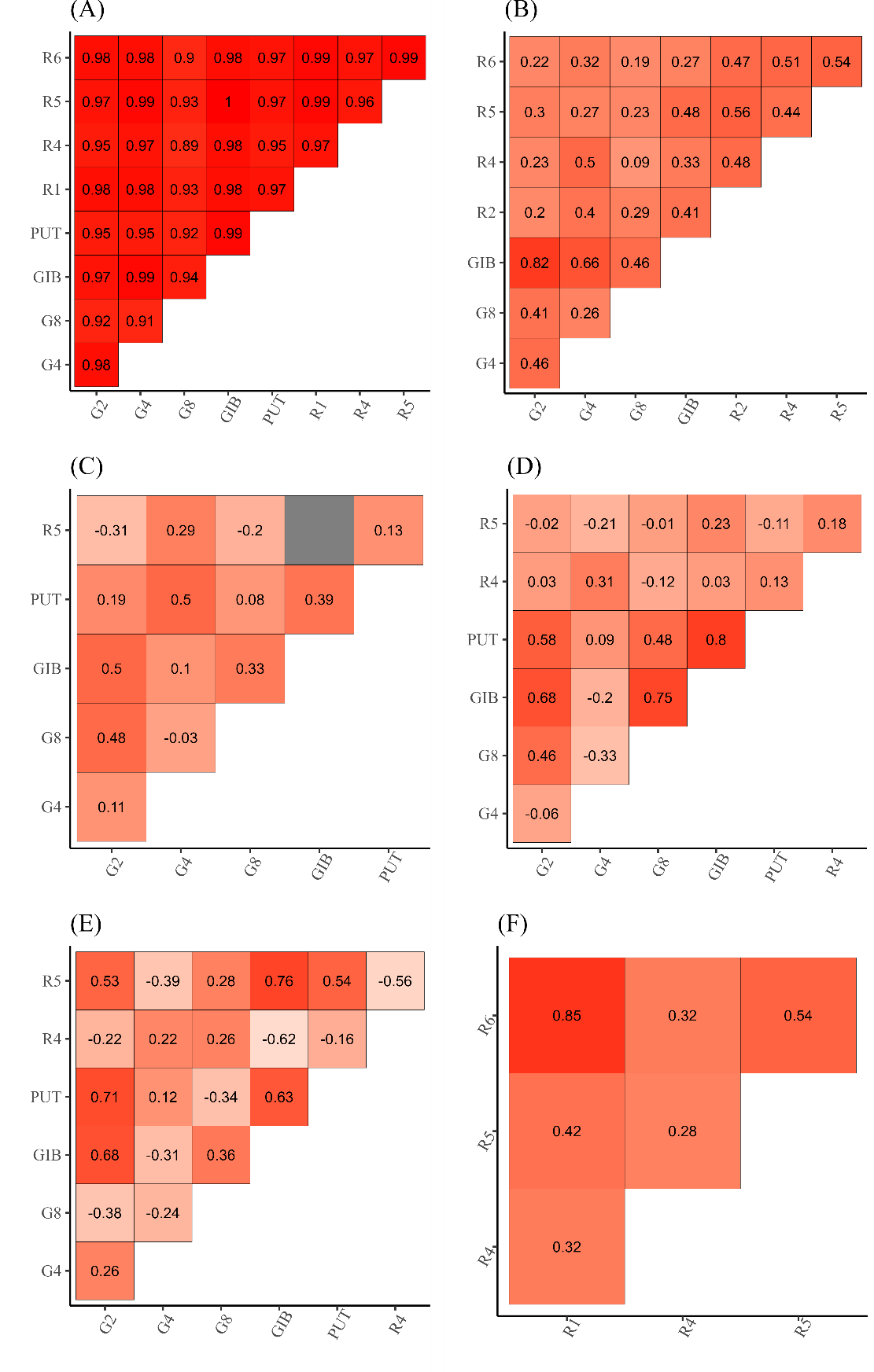


**Figure S1. Pearson Correlation matrices from May-October, 2021,** displaying temperature (A), dissolved oxygen (B), turbidity (C), chlorophyll (D), phycocyanin (E), and Brunt-Väisälä frequency (F).


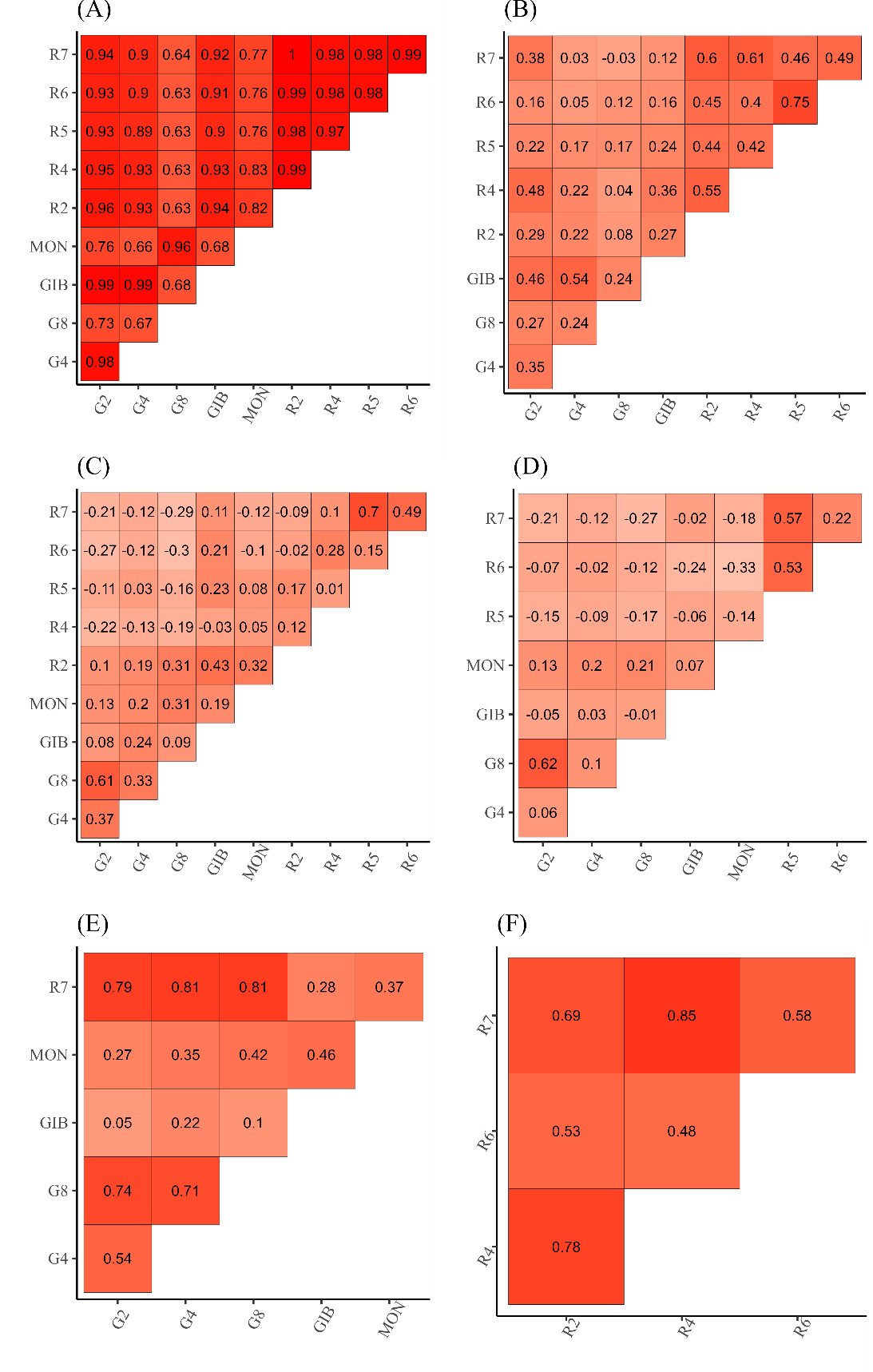


**Figure S2. Pearson Correlation matrices from May-October, 2022,** showing temperature (A), dissolved oxygen (B), turbidity (C), chlorophyll (D), phycocyanin (E), and Brunt-Väisälä frequency (F).


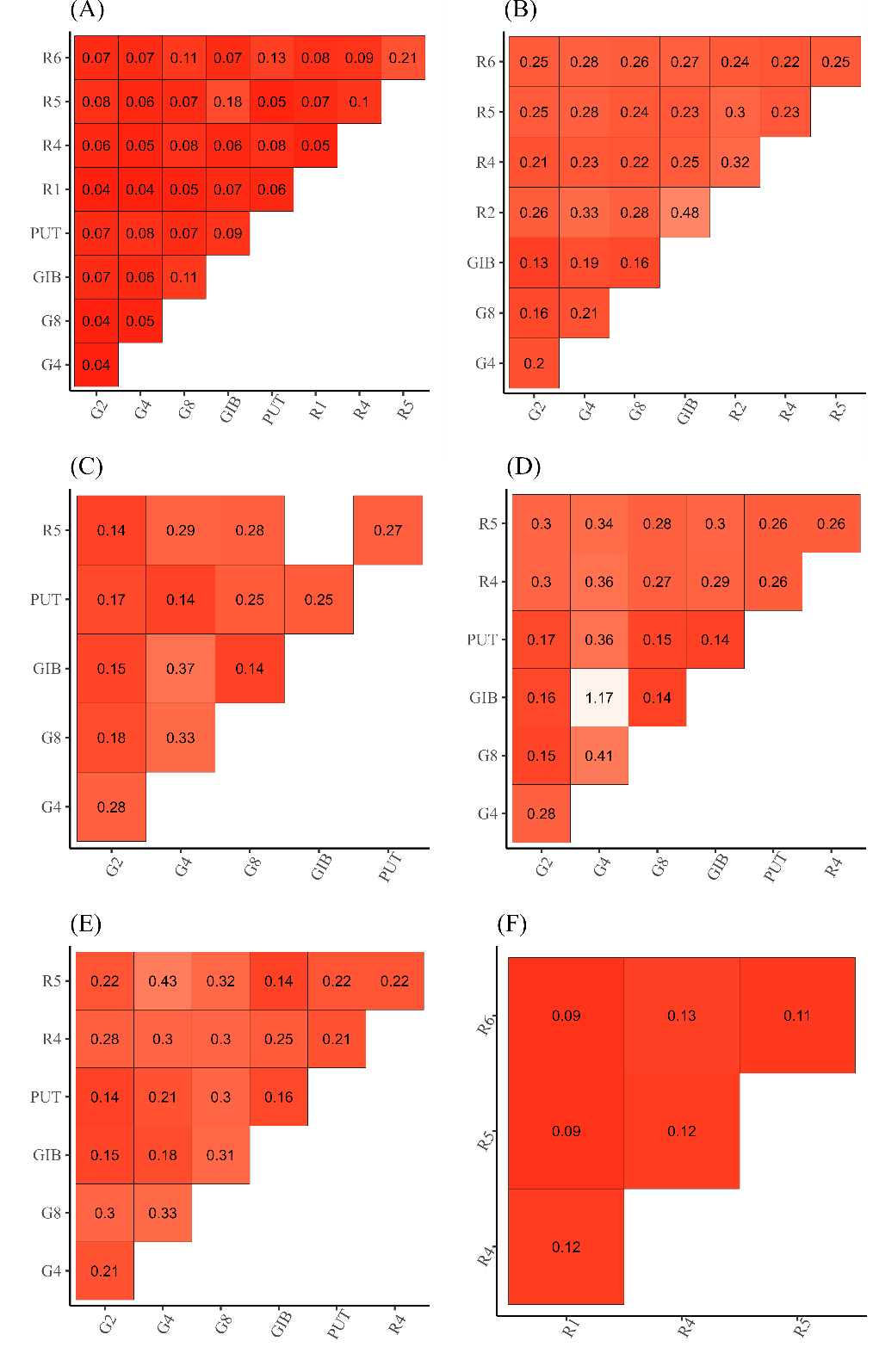


**Figure S3. Matrices with normalized DTW distance from May-October, 2021**, showing temperature (A), dissolved oxygen (B), turbidity (C), chlorophyll (D), phycocyanin (E), and Brunt-Väisälä frequency (F).


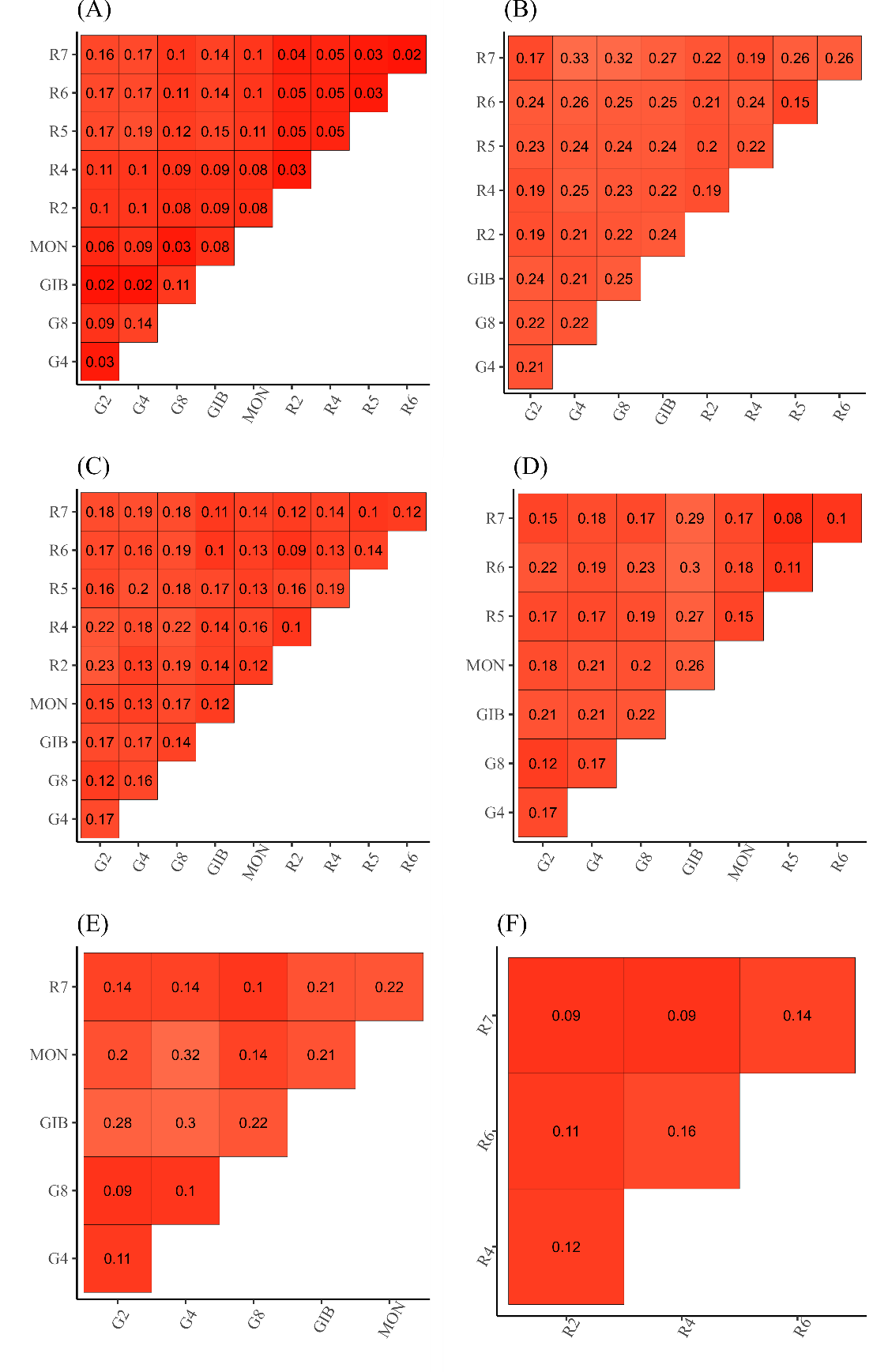


**Figure S4. Matrices with normalized DTW distance from May-October, 2022,** showing temperature (A), dissolved oxygen (B), turbidity (C), chlorophyll (D), phycocyanin (E), and Brunt-Väisälä frequency (F).


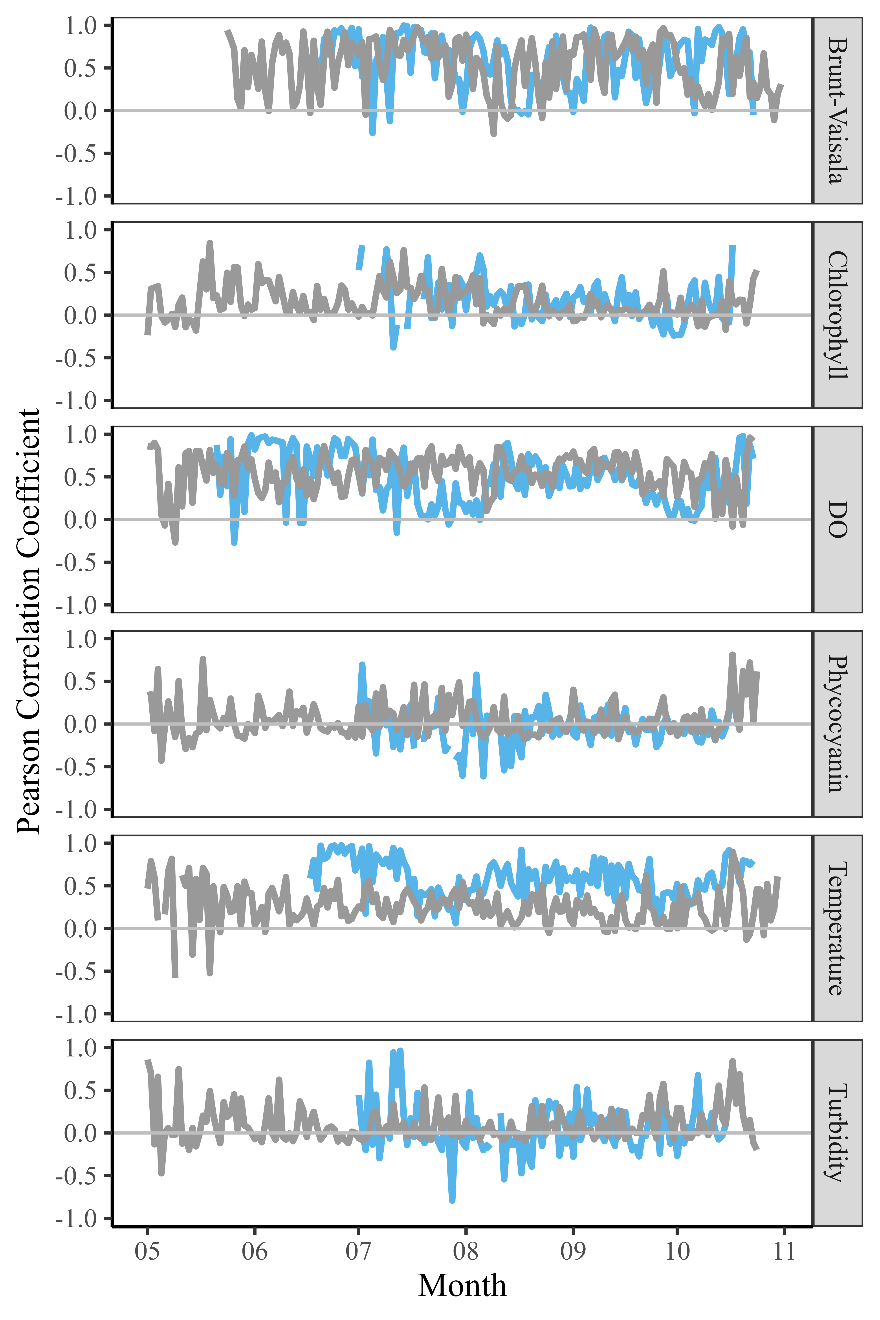


**Figure S5. Daily correlation coefficient for limnological parameters in 2021 (blue) and 2022 (grey).** Correlations were calculated for each pairwise buoy combination then averaged to yield a final daily correlation value.


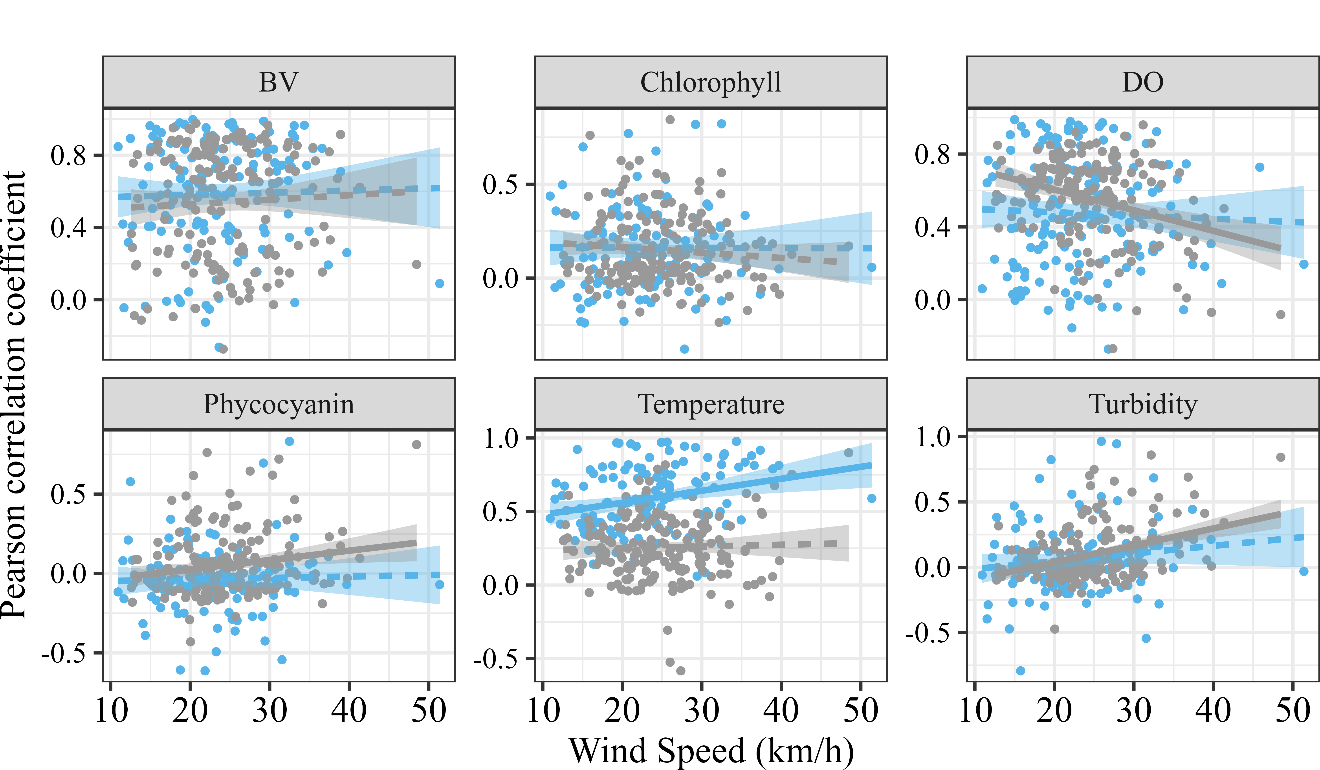


Figure S6. Linear regressions showing correlation as a function of wind speed in 2021 (blue) and 2022 (grey).
